# An interspecies conserved motif of the mouse immune system-released activating agent (ISRAA) induces proliferative effects on human cells

**DOI:** 10.3892/mmr.2014.2225

**Published:** 2014-05-08

**Authors:** SAFA TAHA, MOHAMED DAHMANI FATHALLAH, MOIZ BAKHIET

**Affiliations:** 1 Department of Molecular Medicine, Princess Al-Jawhara Center for Genetics and Inherited Diseases, Arabian Gulf University, Manama 26671, Bahrain; 2 Biotechnology Program, College of Graduate Studies, Arabian Gulf University, Manama 26671, Bahrain

**Keywords:** apoptosis, immunosuppression, proliferation, tumor necrosis factor receptor 1

## Abstract

We have recently described an immune system-released activating agent (ISRAA) as a nervous system-induced factor that stimulates immune responses in the mouse spleen. However, the human ISRAA has not yet been identified. In this study, we examined the effects of the mouse ISRAA protein on human peripheral blood mononuclear cells (PBMCs), to observe if the biological activity of this molecule is consistent between the two different species. Mouse ISRAA demonstrated dose-dependent dualistic effects on human cells, as 5 μg exhibited positive apoptosis and 50 pg exhibited significant proliferation (P<0.05). Furthermore, immunosuppressed cells from patients undergoing immunosuppressive therapy demonstrated significant proliferation to 50 pg ISRAA (P<0.05). Studies to compare sequences in different species revealed a preserved motif, exhibiting 72% similarity with the interspecies conserved signal peptide motif of tumor necrosis factor receptor 1 (TNFR1). A mutant ISRAA lacking this motif was produced and tested for its biological effects. The mutant ISRAA demonstrated neither apoptotic nor proliferative effects compared with wild type. Therefore, an interspecies conserved domain of ISRAA constitutes the active site of the molecule, and its effects on immunocompromised cells should be investigated for future therapies in the treatment of immunosuppressive disorders.

## Introduction

The mechanisms involved in the communication between the immune and nervous systems of the human body remain widely debated and highly controversial. The innate immune system is the first line of the host defense system against invading microbial pathogens, including bacteria, fungi and viruses. By stimulating phagocytosis or the production of cytokines, innate immune cells, including macrophages and dendritic cells (DCs), facilitate the eradication and elimination of these pathogenic microorganisms (1–4). The mechanisms involved in these processes of innate immunity were considered to be nonspecific. However, several recent studies have demonstrated that the nervous system may be a critical regulator of immune responses (5,6). In addition, recent data identified that a neurokinin-2 receptor (NK2R)-dependent neuropeptide signaling pathway was involved in the regulation of antigen-specific T-cell responses, via the activation of DCs, and thus the adaptive immune system may also be subject to the same neuroimmune control (6). In one particular study, the CNS exhibited a highly organized innate immune reaction in response to systemic bacterial infection and cerebral injury (7) and it appears that microglial cells function as sentinels for these innate immune responses (8). Furthermore, intrinsic neuronal innate immune molecules may also function in neurodegenerative processes (9).

In the context of these neuroimmune interactions, in a recent study we described a novel nervous system-induced immune system-released activating agent (ISRAA)(10). It was revealed that this mediator is a signaling factor between the nervous system and the spleen following an immune challenge in mice. The gene which is involved in this process was identified in the mouse system, further cloned and its sequence mapped to chromosome 14 (GenBank accession no. EU552928). The molecular mass of ISRAA was found to be ~15 kDa, along with an ability to activate immunosuppressed cells to proliferate, thus, it is has been suggested that ISRAA is a possible candidate to treat immunosuppressed patients. Furthermore, its mechanism of action may add to the understanding of how the innate immunity may function (10). The human counterpart of the mouse ISRAA is not known, therefore, this study examines the possible immune stimulating effects of the mouse ISRAA on human cells.

## Materials and methods

### Identification and production of wild type and mutated ISRAA

Recombinant wild type ISRAA used in this study was obtained as described previously (10). Mutated ISRAA was synthesized by site directed mutagenesis in which the predicted active site was deleted from the ISRAA gene and cloned in a pUC57 vector. The protein was expressed in a pQE32 vector, purified and then the activity of the mutated ISRAA was tested on PBMCs. The wild type and mutated ISRAA used in the biological assays were endotoxin neutralized using polymyxin B (Sigma, St. Louis, MO, USA).

### Preparation of human peripheral blood mononuclear cells (PBMCs)

#### Blood donors

The study was approved by the Ethics Committee of the Arabian Gulf University, Manama, Bahrain. Blood samples were obtained from healthy donors. None of the participants reported any history of acute or chronic medical problems. For the studies on immunosuppressed cells, blood samples were obtained from kidney transplanted patients. Subjects aged 16–60 years were enrolled in the study and all participating patients were from Salmaniya Medical Centre (SMC; Arabian Gulf University). Patients were undergoing immunosuppressive therapy with <4,000 WBCs/cubic mm and low total lymphocytes count. Written informed consent was obtained from all subjects. EDTA-blood was obtained and PBMCs were isolated according to the techniques described below.

#### Media and reagents

All chemical reagents and media components were obtained from Sigma, unless otherwise noted. The cell culture medium consisted of RPMI-1640 supplemented with 10% heat-inactivated fetal bovine serum (FBS), 10 mM HEPES buffer, 2 mM L-glutamine, 100 U of penicillin/ml and 100 mg of streptomycin/ml. PBMCs were isolated from the blood by centrifugation through Ficoll-Hypaque solution (Histopaque-1077).

#### Isolation of PBMCs

The Ficoll-Hypaque method was utilized in our study to isolate PBMCs from the blood. Briefly, the blood was first diluted 1:1 with phosphate-buffered saline (PBS), and 10 ml of the diluted blood was carefully layered onto a 3 ml Ficoll-Hypaque Plus cushion (Pharmacia Biotech, Uppsala, Sweden) in a tube which was centrifuged at 400 × g for 30 min at 18–20°C. The interface (containing mononuclear cells) was carefully collected and washed twice with PBS and once with RPMI-1640 medium (Life Technologies, Grand Island, NY, USA) containing 1% FBS. The cell pellet was resuspended in 5 ml culture medium (RPMI-1640 medium containing 10% heat-inactivated FBS, 100 IU/ml penicillin G, 100 μg/ml streptomycin, 10 mM HEPES buffer and 2 mM L-glutamine). The viability of mononuclear cells was confirmed by the trypan blue exclusion test. The viable mononuclear cell numbers were counted with a hemocytometer. The viability of mononuclear cells was routinely >95%.

### Cell proliferation assay

Cell Counting kit-8 (CCK-8; Dojindo Laboratories, Kumamoto, Japan) was used for the determination of the number of viable cells in cell proliferation and cytotoxicity assays. Following harvesting, the mononuclear cells were rapidly plated onto a 96-well tissue culture plate (Falcon 3001) at a concentration of 1×10^5^ cells in 100 μl/well and incubated at 37°C in an incubator with 5% CO_2_ for 24 h. The cells were treated with mouse ISRAA protein at concentrations ranging from 5 mg to 1 pg per well. Each concentration was used in triplicate, while 5 μg of phytohemagglutinin (PHA) was used as a positive control, also in triplicate wells. Cells alone were used as a negative control. Plates were incubated for various lengths of time (e.g. 6, 12, 24 and 48 h) in the incubator at (37°C, 95% air, 5% CO_2_ and 100% humidity). Then, 10 μl of CCK-8 solution was added to each well and incubated for a further 4 h. Finally, the absorbance was measured at 450 nm using a microplate ELISA reader (Anthos 2010; Biochrom, Cambridge, UK).

### Apoptosis assay

*In situ* cell death detection kit, Fluorescein (catalog no. 11684795910; Roche Diagnostics GmbH, Mannheim, Germany) was used in this study to determine apoptosis of cells under the effect of different concentrations of the ISRAA protein. This method has also been termed TUNEL (terminal deoxynucleotidyl transferase (TdT)-mediated dUTP-X nick end labeling). The procedure involved culturing and treating PBMCs (1×10^6^/ml) or a cell line (1×10^5^/ml) with different amounts of ISRAA (5 μg and 50 pg/well), then incubating at 37°C in 5% CO_2_ for 24 h. The cell suspension of each sample was fixed by 4% paraformaldehyde for 1 h at room temperature, then washed and permeabilized with 0.1% Triton X-100 and 0.1% sodium citrate in water, freshly prepared for 2 min on ice. The TUNEL reaction mixture (50 μl) was added to the samples and incubated for 60 min at 37°C under wet conditions, protected from light. The TUNEL reaction mixture contained 45 μl equilibration buffer, 5 μl FITC-12-dUTP and TdT, freshly prepared. The label solution alone (50 μl), without any TdT, was added to the negative control to ensure a homogeneous dispersal of TUNEL reaction mixture across the cell monolayer and to avoid loss by evaporation. For positive controls, fixed and permeabilized samples were treated with 5 U μl^−1^ DNase I for 10 min at 37°C, prior to labeling, to induce DNA strand degradation. All samples were washed three times with PBS for 2 min each time and then analyzed by fluorescence microscopy.

### MTT cell proliferation and cytotoxicity assay

The MTT cell proliferation assay (catalog no. 30-1010K-ATCC; Manassas, VA, USA) was used to measure the cell proliferation rate. The assay was performed to study the cytotoxicity effect of ISRAA on the cell lines according to the manufacturer’s instructions. Cell suspension was harvested by centrifugation and resuspended at 1×10^6^/ml. Serial dilutions of ISRAA in culture medium were prepared from 50 μg to 10 pg/ml. The cell suspension (100 μl) was plated in each well in triplicate and three control wells of medium alone (to provide the blanks for absorbance readings) were included, in addition to another three wells with cell suspension alone as a negative control.

Each serial dilution of ISRAA protein (10 μl) was added to each well in triplicate. Plates were incubated at 37°C in 5% CO_2_ for 24, 48 and 72 h. Later on, 10 μl of 5 mg/ml MTT reagent was added to each well, including the controls and incubated for 4 h at 37°C. Following this, 100 μl of the MTT solvent (acidic isopropanol 0.04 M HCl in absolute isopropanol) was added to all wells including controls. Plates were covered with tinfoil and agitated on an orbital shaker for 15 min. Finally, the absorbance at 690 nm was recorded and the cytocide rate was calculated from the below formula: Cytocide rate (%) = (OD control − OD experiment)/OD control × 100.

### Staining of the proliferation marker Ki-67 in human PBMCs

Ki-67 proliferation marker was used in our study to determine the effect of ISRAA on human cells. PBMCs of healthy donors were prepared using density gradient centrifugation method as described previously. Cells were washed 2 times in PBS, counted by hemocytometer chamber, number of cells adjusted to 1×10^6^/ml and then cultured in complete RPMI-1640 medium, supplemented with 10% FCS, penicillin/streptomycin and glutamine in tissue culture slides. Cells (100 μl/well) were stimulated with two different concentrations of ISRAA protein (5 μg and 50 pg). For negative control, resting cells was used (unstimulated PBMC) and for positive control, cells were stimulated with 5 μg PHA. Cells were incubated at 37°C, 5% CO_2_ for 24 h.

### Preparation of cytospin from single cell suspension

The cytospin slides were prepared mounted with the paper pad and the cuvette in the metal holder. Cell suspensions (100 μl) were loaded into each cuvette and spun at 16.582 × g for 3 min. The slide, paper and cuvette were extracted without disarranging. The cuvette and the paper were carefully detached without damaging the fresh cytospin. Immediately, slides were fixed in 4% paraformaldehyde for 1 h and washed in PBS (3 times, 5 min). Then, cells were permeabilized in 0.25% Triton X-100 for 10 min followed by incubation in a moist chamber with the primary antibody (Rabbit anti-human Ki-67, Abcam-ab15580-UK) at a dilution of 1:200 for 1 h at room temperature following a PBS rinse for 5 min. Subsequently, incubation with the secondary antibody (anti-rabbit IgG, 1:500, ABC Elite kit; Vector Laboratories, Burlingame, CA, USA) was performed for 30 min, followed by avidin-biotin amplification (ABC kit-DAKO) for 30 min. The detection reaction was conducted with 0.1% 3′3′-diaminobenzidine (DAB-DAKO) and counterstaining with hematoxylin.

### Statistical analysis

Mann-Whitney’s test was used to calculate the level of statistical significance (^*^P<0.05, ^**^P<0.005, ^***^P<0.0005). P<0.05 was considered to indicate a statistically significant difference.

## Results

### Effects of ISRAA on human PBMCs

The biological effects of mouse ISRAA were tested on human PBMCs to examine the existence of a possible shared interactive conserved domain. Accordingly, human PBMCs from healthy donors (10^5^ cells in 100 μl of complete medium/well) were treated with different concentrations of ISRAA ranging from (5 μg-1 pg) and cultured for 24, 48 and 72 h. Then, the following biological assays were recorded.

### Cell proliferation

**Figure 1:**
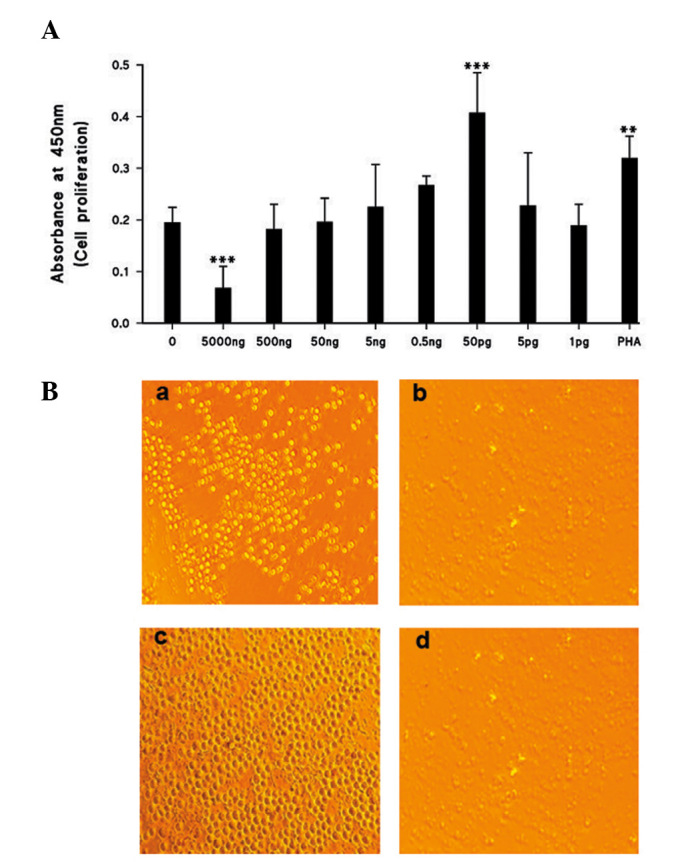
Effects of ISRAA on hPBMCs. (A) PBMCs from healthy donors (100 μl of 1×10^6^ cells/well) were treated with different concentrations of ISRAA (5,000 ng-1 pg) and the proliferation of cells was monitored by MTT assay. Cells treated with 50 pg ISRAA demonstrated significant proliferation compared with those treated with 5,000 ng. PHA was used as a positive control while cells alone were used as a negative control. Mann-Whitney’s test was used to calculate the level of significance (^**^P<0.005, ^***^P<0.0005). All experiments were repeated >6 times. (B) Images (magnification, ×20) captured by an inverted microscope demonstrated the proliferative response of hPBMCs cultured for 24 h. (a) Unstimulated cells as a negative control; (b) cells stimulated with PHA as a positive control; (c) cells stimulated with 50 pg ISRAA; (d) cells treated with 5000 ng ISRAA. All experiments were repeated >6 times. ISRAA, immune system-released activating agent; hPBMCs, human peripheral blood mononuclear cells; CCK, cell counting kit; PHA, phytohemagglutinin.

As demonstrated in Fig. 1A and B, a significant reduction (P<0.0005) in the number of proliferating cells was registered with the highest concentration of ISRAA used (5 μg; 500 ng). With further dilutions of ISRAA, significant proliferation was recorded with 50 pg (P<0.0005) compared with non-stimulated cells. PHA was used as a positive control and demonstrated significant proliferative effects (P<0.005).

### Apoptosis and cytotoxic effect

**Figure 2:**
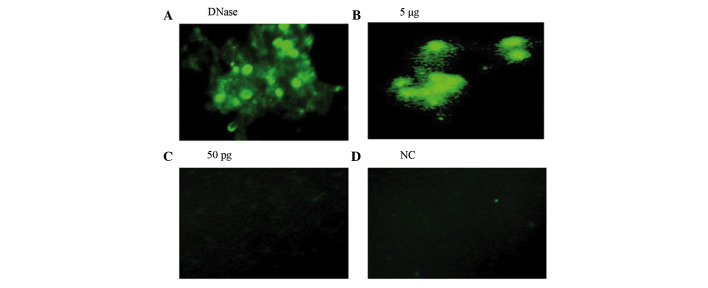
Cytotoxic effect of ISRAA on hPBMCs. The cytotoxic effect of 5 μg of ISRAA on hPBMCs was monitored by an *in situ* cell death assay in which cleavage of genomic DNA during apoptosis yields double strand as well as single strand breaks (nicks), and then was identified by labeling free 3′-OH terminal with fluorescein. Cells treated with (B) 5 μg ISRAA demonstrated apoptosis of the cells compared with the effect of (C) 50 pg ISRAA on hPBMCs and to (A) the positive control treated with DNase enzyme, while in (D) the NC, the cells were incubated with label solution only (without terminal transferase). Experiments were repeated >6 times. ISRAA, immune system-released activating agent; hPBMCs, human peripheral blood mononuclear cells; NC, negative control.

The cytotoxic effect of ISRAA on human PBMCs (1×10^5^/100 μl culture medium) was determined by an *in situ* cell death assay in which 5μg of ISRAA demonstrated more apoptosis of the cells compared with 50 pg of ISRAA and the positive control which was treated with DNase (Fig. 2).

### Proliferation marker Ki-67

**Figure 3:**
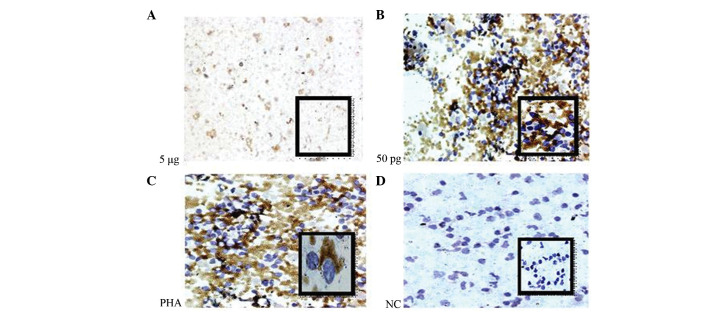
Ki-67 immunostaining. Ki-67 immunostaining in cells treated with (A) 50 μg ISRAA, revealing nuclear negativity of cells compared with (B) brown granular nuclear reactivity in cells treated with 50 pg (hematoxylin counterstain). (C) Positive control in which cells were treated with 5 μg PHA, (D) represents the NC in which cells are at resting stage (non-stimulated cells; magnification, ×20 and ×40). Experiments were repeated >6 times. ISRAA, immune system-released activating agent; hPBMCs, human peripheral blood mononuclear cells; CCK, cell counting kit; PHA, phytohemagglutinin; NC, negative control.

Staining and detection of the proliferation marker Ki-67 revealed that its highest expression was exhibited by the cells treated with 50 pg (Fig. 3B) compared with the cells treated with 5 μg of ISRAA (Fig. 3A. Cells stimulated with 5 μg PHA were used as a positive control (Fig. 3C) and cells alone without treatment used as a negative control (Fig. 3D).

### Effects of ISRAA on immunosuppressed cells

**Figure 4:**
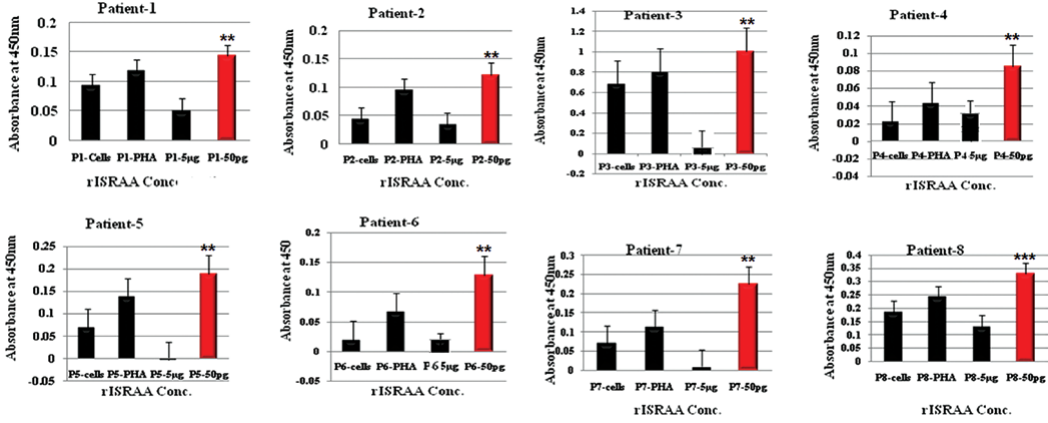
Effect of ISRAA on immunosuppressed cells. This figure illustrates the effects of 5 μg and 50 pg of ISRAA on patient’s PBMCs in terms of cell proliferation measured by an MTT assay. Note significant proliferative effects in all patients with 50 pg of rISRAA (^**^P<0.005, ^***^P<0.0005). PHA did not reveal significant proliferative effects compared with non-stimulated cells. rISRAA, recombinant immune system-released activating agent; PBMCs, peripheral blood mononuclear cells; PHA, phytohemagglutinin; Conc, concentration.

To examine the effects of different concentrations of ISRAA on immunosuppressed cells, blood samples from eight kidney-transplanted patients undergoing immunosuppressive therapy were obtained, and the biological activity of ISRAA on the cells, as measured in terms of cell proliferation, was monitored. Results demonstrated significant proliferation (P<0.005) following stimulation with 50 pg ISRAA in all of the examined patients. However, the 5 μg ISRAA cells exhibited cell proliferation levels below that observed in the unstimulated cells (controls). PHA stimulated cells were used as a positive control, however no significant proliferation was noted. Untreated cells were used as a negative control in all experiments (Fig. 4).

### Active site (motif) of the ISRAA gene

**Figure 5:**
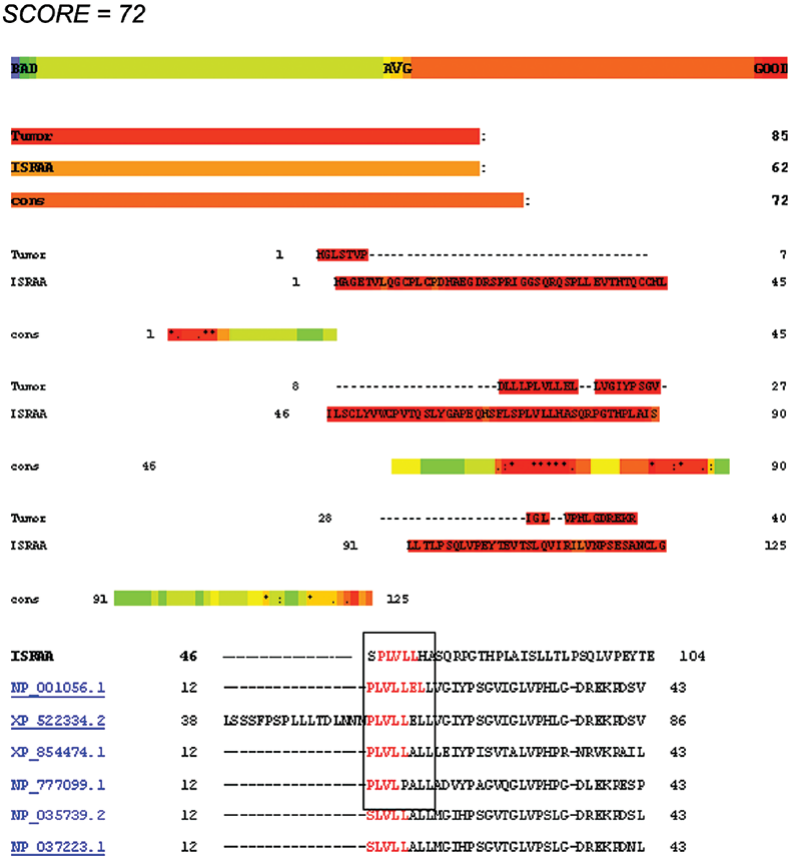
Alignment of ISRAA with TNFR1. The alignment of ISRAA with the SP motif of TNFR1 using T-Coffee Tool to determine the conserved regions revealed 72% similarity. ISRAA, immune system-released activating agent; SP, signal peptide; TNFR1, tumor necrosis factor receptor 1.

**Figure 6:**
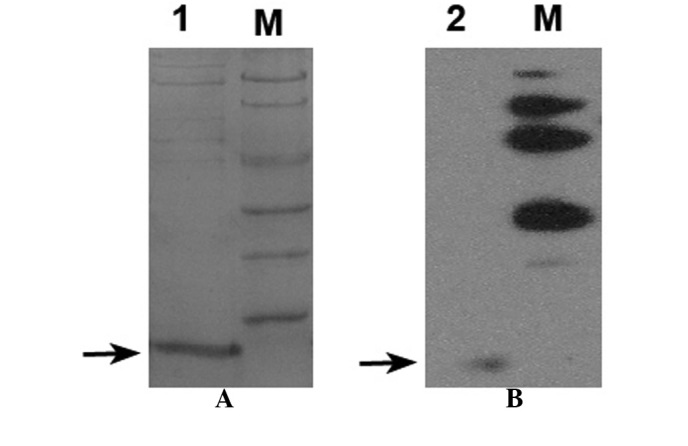
Mutated ISRAA gene. Mutated ISRAA was synthesized by site directed mutagenesis in which the predicted active site was deleted from the ISRAA gene, cloned in an pUC57 vector, protein was expressed in pQE32 vector, purified and then the activity of the mutated ISRAA was tested on hPBMCs. Protein sequence, isoelectric point (PI) and molecular mass (MW) are demonstrated as follows: PI/MW; 7.31/11955.0. Protein sequence: MRGSHHHHHHHGIRMAGETVLQGCPLCPDH AEGDRSPRIGGSQRQSPLLEVTHTQCCHLILSCLYVWCPVTQSLYGA PEQHSYTEVTSLQVIRILVNPSESANCLGKL. SDS-PAGE analysis of the mutated protein: (A) Lane 1, SDS-PAGE (4–20% gradient gel) of ISRAA-mutant protein (3 μg). (B) Lane 2, western blotting checking the mutated protein using anti-His antibody. Arrows indicate 11.955 kDa, the mutated protein on the SDS-PAGE and western blot analysis. Wild type ISRAA = 15 kDa. ISRAA, immune system-released activating agent; M, protein marker; hPBMCs, human peripheral blood mononuclear cells.

The SP (signal peptide) motif of TNFR1 (tumor necrosis factor receptor 1) was identified to have an interspecies conserved motif between rats, mice and humans (Table I) transmitting signals for either cell death or proliferation, depending on the concentration of the effector molecule, as recorded by ISRAA effects. The alignment of ISRAA with the SP motif of TNFR1 revealed 72% similarity (Fig. 5). Accordingly, mutant ISRAA lacking this motif was constructed, the protein sequence, isoelectric point and molecular mass of mutated ISRAA were determined as demonstrated in Fig. 6.

**Figure 7:**
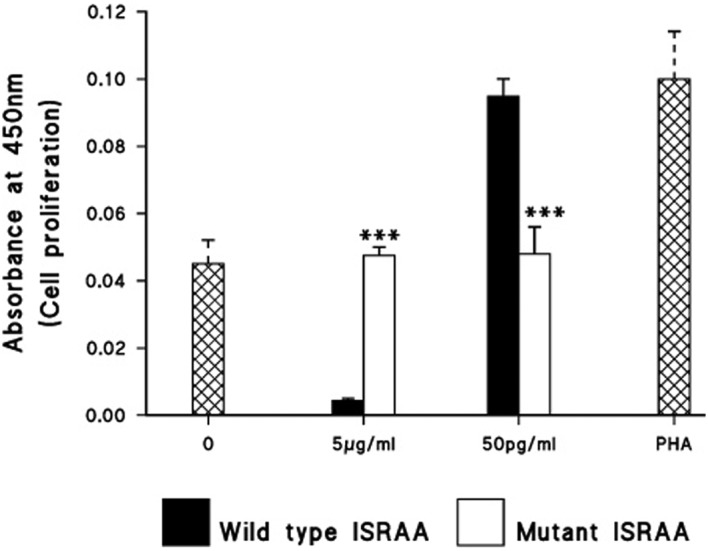
Effects of mutated ISRAA on human PBMCs. PBMCs from healthy donors (100 μl of 1×10^6^ cells/well) were treated with 50 pg and 5 μg of wild type and mutated ISRAA, and proliferation of cells was determined by CCK-80 assay. Cells treated with 50 pg wild ISRAA demonstrated significant proliferation, an effect which was abrogated by mutation of the predicted active site of ISRAA (P<0.0005). Also, the cytotoxic effect of the 5 μg wild type ISRAA was significasntly inhibited (P<0.0005). PHA was used as a positive control while cells alone as a negative control. Experiments were repeated >6 times. ISRAA, immune system-released activating agent; hPBMCs, human peripheral blood mononuclear cells; CCK, cell counting kit; PHA, phytohemagglutinin.

**Figure 8:**
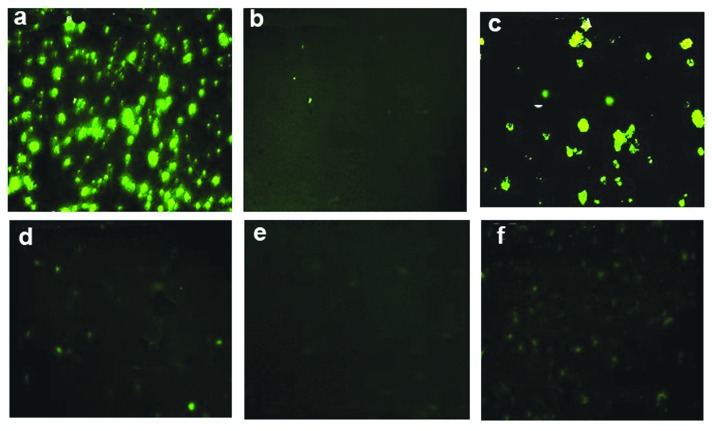
Cytotoxic effect of mutated ISRAA on hPBMCs. Cytotoxic effect of 5 μg of mutated ISRAA on hPBMCs was detected by an *in situ* cell death assay in which cleavage of genomic DNA during apoptosis yields double strand, as well as single strand breaks (nicks), and then was identified by labeling free 3′-OH terminal with fluorescein. (a) Positive control cells treated with DNase enzyme, (b) negative control cells incubated with label solution only (without terminal transferase). (c) Cells treated with 5 μg wild type ISRAA demonstrated apoptosis of the cells, an effect which was lost upon (d) mutation of ISRAA predicted active site. Micrographs (e) and (f) represent 50 pg wild type and mutant ISRAA, respectively where no cell death was recorded. Experiments were repeated >6 times. ISRAA, immune system-released activating agent; hPBMCs, human peripheral blood mononuclear cells.

The MTT assay and TUNEL test were used, respectively, to determine the proliferation rate and *in situ* cell death of the mutant ISRAA and its wild type. As illustrated in Fig. 7 and Fig. 8, mutation of the predicted ISRAA active site resulted in significant loss of proliferative and cytotoxic function produced by the 50 pg (proliferation) and 5 mg (cytotoxicity), as compared with the wild type ISRAA (P<0.0005; Fig. 8).

## Discussion

In the present study, the results demonstrated the potential proliferative effects of the mouse ISRAA on human cells, as mediated by an interspecies conserved motif. Titration of the mouse ISRAA activity on human cells revealed potent dose-dependent dualistic effects. The effects of ISRAA were variable at different concentrations, as revealed by the data demonstrating that the high concentration (5 μg) induced apoptosis and the low concentration (50 pg) triggered cell activation. This variation in response may result from the differential expression of multiple receptors on the cells that bind to ISRAA, with variable affinity, which activates a series of different signal transduction cascades and ultimately produces cellular effects that are dependent on specific receptor interactions. High concentrations of ISRAA allows rapid, high affinity binding to receptor death domains, since this was the observed prominent effect of such a concentration. Dilution of ISRAA to a lower concentration may have prompted the dissociation of ISRAA-death domain receptor complexes and generated the optimal conditions stimulating its subsequent binding to other receptors, that induced intracellular signaling pathways for cell activation and survival. These types of receptors are able upon stimulation to form clusters with the same group of signaling proteins (11). Curiously, however, the ligation of these receptors is able to promote two different cell fates; proliferation or death. Also, ligands and receptor bindings are considered to be critical regulators of cell proliferation, differentiation and apoptosis. As a result, targeting different signaling transduction pathways has been a focal point in the development of novel treatment strategies in cancer therapeutics (12,13).

There are existing situations in which the same stimulus, mediated by the same member of the TNF receptor family, triggers either proliferation or apoptosis (14,15). Such aberrant behavior is explained by the fact that these two cellular processes, although occurring through similar initial pathways, participate in signal bifurcation (11,16), so that each of these receptors transmits one signal eliciting cell death and another that induces proliferation. This dichotomic signalization is dictated by the nature of the effector molecules recruited by the receptors, and the final cellular output depends on the relative frequency of these two signalization events. Thus, ISRAA is either capable of binding to different receptors to induce the same or different signals, or may bind to a receptor that is able to active different intracellular pathways leading to either survival or death, which is dependent on the concentration of ISRAA as an effector molecule. Furthermore, the alignment of ISRAA with the SP motif of TNFR1, which demonstrated 72% interspecies similarity between rat, mouse and human (17), may explain the dose-dependent dualistic effects of mouse ISRAA on human cells.

ISRAA had a marked effect on the immunosuppressed cells obtained from patients undergoing immunosuppressive therapy. Prior to ISRAA stimulation, a number of these cells completely failed to proliferate following mitogen stimulation. However, a low dose of ISRAA elicited significant proliferative responses in all of the examined patients cells. Variations in proliferative responses were observed between the patients’ samples, which may be explained by a number of different parameters including age, sex, patient history, immunosuppressive drug used and more. Regardless of the variation among patients, all of the cells demonstrated a significant response to ISRAA.

In conclusion, ISRAA is an immune mediator produced as a result of a nerve stimulus initiated by immune challenge. The active site of ISRAA constitutes an interspecies conserved motif, sharing 72% homology with TNFR1, a receptor connected to intracellular domains that induce dose-dependent signals for survival or death. The demonstrated proliferative stimulatory effects of ISRAA on immunosuppressed cells should be investigated in future therapeutic approaches in the treatment of immunosuppressive diseases.

## Figures and Tables

**Table I:** HomoloGene table demonstrating TNFR1 and SP motif in different organisms.

Protein acc.	Gene	Organism
NP_001056.1	*TNFRSF1A*	*H. sapiens*
XP_522334.2	*TNFRSF1A*	*P. troglodytes*
XP_854474.1	*TNFRSF1A*	*C. lupus*
NP_777099.1	*TNFRSF1A*	*B. taurus*
NP_035739.2	*TNFRSF1A*	*M. musculus*
NP_037223.1	*TNFRSF1A*	*R. norvegicus*

TNFR1, tumor necrosis factor receptor 1; SP, signal peptide; Acc, accession number.
